# Unusual presentation of a giant congenital melanocytic nevus

**DOI:** 10.11604/pamj.2026.53.94.50505

**Published:** 2026-02-23

**Authors:** Mayur Pradip Mhaske, Amol Deshpande

**Affiliations:** 1 Department of Rachana Sharir, Mahatma Gandhi Ayurved College Hospital and Research Centre, Datta Meghe Institute of Higher Education and Research (Deemed to be University), Salod (H), Wardha, India

**Keywords:** Hyperpigmented hairy plaque, melanocytic nevus, giant congenital nevus

## Image in medicine

A 38-year-old patient presented with a large, asymptomatic, hyperpigmented plaque over the lower back, which had been present since birth. There was no history of pain, itching, discharge, ulceration, or recent change in size or color. The patient had no associated systemic illness and no personal or family history of malignancy. Cutaneous examination revealed a well-demarcated, dark brown to black hairy hyperpigmented plaque measuring approximately large in size, occupying the lower lumbar region. The surface was densely covered with coarse terminal hairs, giving a characteristic “hairy” appearance. The surrounding skin was normal. Based on the congenital onset and clinical morphology, the provisional diagnosis of giant congenital melanocytic nevus was considered. The important differential diagnoses included Becker nevus, epidermal nevus, and plexiform neurofibroma. Congenital melanocytic nevi are benign proliferations of melanocytes present at birth and are classified based on size. Giant variants are rare and carry an increased lifetime risk of malignant melanoma, particularly when larger than 20 cm in diameter. Although the present lesion is asymptomatic with no alarming features, long-term surveillance is essential. Surgical excision, laser therapy, or staged reconstruction procedures may be considered for cosmetic reasons or risk reduction.

**Figure 1:**
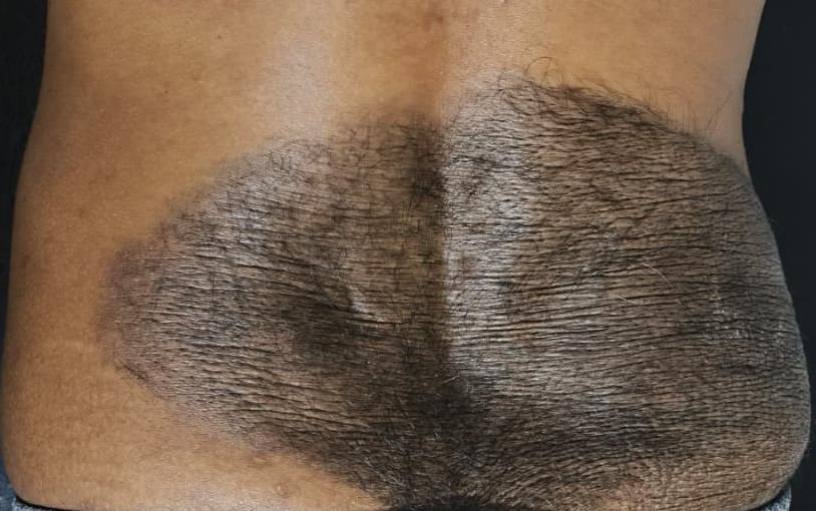
large congenital hyperpigmented hairy plaque over the lower back, consistent with a giant congenital melanocytic nevus (side view)

